# Exploring trends and predictors of long-term asthma remission^∗^

**DOI:** 10.1016/j.waojou.2025.101127

**Published:** 2025-10-03

**Authors:** Vera Veith, Frauke Pedersen, Henrik Watz, Anne-Marie Kirsten, Folke Brinkmann, Matthias V. Kopp, Anna-Maria Dittrich, Gesine Hansen, Nicole Maison, Bianca Schaub, Erika von Mutius, Klaus F. Rabe, Thomas Bahmer, Mustafa Abdo, the ALLIANCE Study Group

**Affiliations:** a LungenClinic Grosshansdorf, Airway Research Center North (ARCN), German Center for Lung Research (DZL), Grosshansdorf, Germany; b Velocity Clinical Research Germany, Ahrensburg, Formerly Pulmonary Research Institute at LungenClinic Grosshansdorf, Airway Research Center North (ARCN), German Center for Lung Research (DZL), Germany; c University Children’s Hospital, Luebeck, Airway Research Center North (ARCN), German Center for Lung Research (DZL), Germany; d Department of Paediatric Respiratory Medicine, Inselspital, University Children’s Hospital of Bern, University of Bern, Bern, Switzerland; e Department of Paediatric Pneumology, Allergology and Neonatology, Hannover Medical School, Hannover, Germany; f Biomedical Research in Endstage and Obstructive Lung Disease Hannover (BREATH), German Center for Lung Research (DZL), Germany; g Department of Paediatric Allergology, Dr von Hauner Children’s Hospital, Ludwig Maximilians University, Munich, Germany; h Comprehensive Pneumology Center, Munich (CPC-M), German Center for Lung Research (DZL), Germany; i Institut für Asthma- und Allergieprävention (IAP), Helmholtz Zentrum Munich, Deutsches Forschungszentrum für Gesundheit und Umwelt (GmbH), Munich, Germany; j University Hospital Schleswig-Holstein-Campus Kiel, Department for Internal Medicine I, Airway Research Center North (ARCN), German Center for Lung Research (DZL), Kiel, Germany; k German Center for Child and Adolescent Health (DZKJ), Dr von Hauner Children’s Hospital, LMU, Munich, Germany

**Keywords:** Asthma, Disease remission, Eosinophils, Predictive value of tests, Type 2 inflammation, Biological therapy, Longitudinal studies, Comorbidity

## Abstract

**Rationale:**

Asthma remission is a state of low to no disease activity. To date, little is known about predictors and the achievability of long-term asthma remission.

**Objective:**

To identify clinical predictors and trends of long-term remission in a cohort of adults with mild to severe asthma.

**Methods:**

This study included 203 adults with mild to severe asthma from the All Age Asthma Cohort, followed over 6 years. Participants attended 5 visits, during which type 2 inflammation markers (blood and sputum eosinophils, fractional exhaled nitric oxide), lung function measurements (oscillometry, spirometry), atopy and systemic comorbidities were assessed. Clinical remission was defined by an Asthma Control Test score of ≥20 plus the absence of both severe exacerbations and systemic corticosteroid use in the past 12 months, and normal or stable lung function. Long-term remission was defined as remission lasting at least 3 consecutive years, while short-term remission lasted 1 or 2 consecutive years.

**Results:**

The frequencies of long-term, short-term, and no remission were 27%, 34%, and 39%, respectively. 16% of all patients with severe asthma achieved long-term remission, compared to 65% of those with mild-to-moderate disease. Over one-third of all patients never achieved remission and had persistent T2 markers despite high-dose ICS. Predictors of no asthma remission included number of persistent T2-markers (OR:0.26, CI: 0.11, 0.61), frequency dependence of resistance (FDR, R5-R20Hz; OR:0.36, CI: 0.15, 0.82), FEV1/FVC (OR:0.16, CI: 0.06, 0.37), GERD (OR:0.23, CI: 0.1, 0.5), CVD (OR:0.44, CI: 0.22, 0.87), dyslipidemia (OR:0.38, CI: 0.13, 1.05), whereas sensitization to house dust mite was associated with a higher remission rate (OR:2.06, CI: 1.03, 4.17). During long-term follow-up, significant adjusted predictors of no remission were sputum eosinophils, small airway dysfunction, and airflow obstruction.

**Conclusion:**

This study highlights a substantial unmet need in achieving long-term remission, particularly in patients with persistent type 2 inflammation and impaired lung function, prompting re-evaluation of targeting T2 inflammation earlier to prevent lung function impairment.

## Introduction

Asthma is a chronic respiratory disease characterized by airway inflammation, hyperresponsiveness, and variable airflow obstruction, and is associated with significant morbidity and impaired quality of life.^1^ Asthma follows a variable course, with some patients experiencing remission with or without the need for permanent treatment. In recent years, achieving remission has emerged as a critical treatment goal in severe asthma management, supported by advances in therapeutic options, including monoclonal antibodies.^2^ Generally, clinical remission is defined as a state of well-controlled disease, characterized by minimal or no symptoms and stable or normal lung function, either with or without medication.^3^

Several studies have examined clinical remission in asthma, primarily focusing on spontaneous remission in childhood or adolescence,^4^^,^^5^ or remission achieved with Type 2 (T2)-targeted therapies in severe cases.^6^^,^^7^ Furthermore, recent research has explored asthma remission over a one-year period in patients with severe asthma.^8^ However, long-term data on asthma remission across the full range of disease severity, from mild to severe asthma, with a comprehensive spectrum of biomarkers remain scarce. Additionally, well-characterized cohorts with data on key asthma predictors such as small airway dysfunction, sputum cell differentiation^9^ and atopic and non-atopic comorbidities are needed to understand the determinants of remission, considering the complexities of asthma management, across diverse patient populations and over long-term follow-up periods. Therefore, this study investigates factors associated with asthma remission across disease severity, with a focus on T2 inflammation, airflow obstruction and small airway dysfunction. Given the role of eosinophilic inflammation in lung function impairment,^9^ we analyzed key T2 markers—sputum eosinophils, blood eosinophils, and fractional exhaled nitric oxide. Considering the fluctuating nature of symptoms, we conducted a 6-year analysis of predictors, including asthma control, medication use, disease severity, lifestyle, and demographics. This comprehensive approach aims to refine remission prediction and inform clinical management.

## Methods

### Study design

We performed a longitudinal analysis utilizing data collected in the adult arm of the multicenter prospective All Age Asthma Cohort (ALLIANCE). This cohort encompasses both pediatric and adult patients with asthma and was established by the German Centre for Lung Research (DZL).^10^ The study received ethical approval from the local ethics committee at Luebeck Medical School (Approval No. Az.21-215) and is registered on ClinicalTrials.gov (adult arm: NCT02419274). Prior to enrollment, written informed consent was obtained from all participants. Since 2014, the adult arm of ALLIANCE has recruited patients with mild to severe asthma, as well as healthy controls. A total of 5 study visits were included in the analysis with a follow-up duration of 6 years. The time interval between the baseline visit (BL) and follow-up visit 1 (FU1), as well as between FU1 and FU2, was 12 months. The intervals between FU2 and FU3, and between FU3 and FU4, were 24 months each. The recruitment process, as well as the inclusion and exclusion criteria, has been described previously.^10^ Patients were required to have stable disease, with no acute exacerbations or airway infections occurring within 4 weeks prior to each study visit. Patients had to attend at least 3 visits to be included in this analysis. The data originate from a single study site.

At each study visit, data were collected on medication use, exacerbations, hospital admissions, and other relevant clinical events occurring within the previous 12 months. Asthma severity was defined based on the GINA treatment steps. Mild-to-moderate asthma was defined as asthma managed with GINA treatment steps I–III, while severe asthma was defined as requiring treatment with steps IV–V.

### Asthma remission

Clinical remission in asthma was defined based on the following criteria: an Asthma Control Questionnaire (ACT) score of 20 or higher, absence of severe exacerbations in the last 12 months, no systemic corticosteroid use in the past 12 months, and normal lung function, defined as a forced expiratory volume in 1 s (FEV1) at or above 80% of the predicted value, or stable lung function, defined as a change in percent predicted FEV1 of less than 5% from the previous visit.^11^ Severe exacerbation is defined as symptom deterioration that requires oral corticosteroids (OCS) for at least 3 days. Remission duration was arbitrary classified as either long-term or short-term. Long-term remission was defined as a remission lasting at least 3 consecutive years, while short-term remission lasted 1 or 2 consecutive years. Remission period can occur at any time after enrollment.

### Lung function measurement

We conducted forced spirometry and impulse oscillometry (IOS) following current recommendations.^12^^,^^13^ The corresponding measures and markers of small airway function included the frequency dependence of resistance (FDR), calculated as the difference in resistance between 5 Hz and 20 Hz (R5Hz - R20Hz), and area under the reactance curve (AX). Both are considered markers of small airway narrowing of asthma patients,^14^^,^^15^ with a threshold of greater than 0.07 kPa/L per s being indicative of small airway dysfunction.^16^^,^^17^ Spirometry measurements included FEV1% predicted, along with the post-bronchodilator FEV1/FVC ratio to evaluate the presence of airway obstruction.

### T2-markers

Type 2 inflammation was evaluated through the measurement of eosinophil levels in blood and sputum, as well as the assessment of fractional exhaled nitric oxide (FeNO). The cutoffs for type 2 inflammation were set at sputum eosinophils ≥2 (%), blood eosinophil count ≥0.15 (/μl), and FeNO ≥20 (ppb).^18^ Sputum induction and processing were carried out in accordance with established standardized operating procedures.^19^

### Physical activity

Physical activity was quantified over a one-week period using a multisensory activity monitor (SenseWear Pro Armband, BodyMedia, Pittsburgh, PA), as detailed in previous studies.^20^ The average number of steps per day was calculated. To ensure the validity of the analyses, a threshold of 94% of wearing time (22.5 h) for a minimum of 5 days was established.^21^

### Statistical analysis

Differences in clinical variables across remission duration groups and asthma severity categories were assessed using one-way analysis of variance (ANOVA) for normally distributed continuous variables, Kruskal–Wallis tests for non-normally distributed continuous variables, and Fisher’s exact test for categorical variables.

Odds ratios (ORs) with 95% confidence intervals (CIs) were calculated from contingency tables to investigate associations between categorical predictors and remission status. Fisher’s exact test was used to determine the significance of these associations.

To identify independent predictors of asthma remission over time, we used a generalized linear mixed-effects model (GLMM) with a binomial distribution and logit link function to model the binary outcome variable remission status (remission vs. no remission). Fixed effects included scaled measures of asthma severity (GINA treatment step), sputum eosinophil percentage, small airway resistance (R5–R20), airflow obstruction (FEV_1_/FVC ratio), age, sex, and body mass index (BMI). Because T2 biomarkers are correlated, we pre-specified a one-T2-marker-per-model approach. To foreground airway-level inflammation, the primary model included sputum eosinophil percentage. In separate models, sputum eosinophils were replaced by FeNO or by blood eosinophil counts. Random intercepts were included for subject and study visit to account for repeated measures and visit-specific variability. Model fitting was conducted using the *glmer* function from the *lme4* package with the *bobyqa* optimizer. A p-value less than 0.05 was considered statistically significant. Statistical analyses were conducted using R version 4.5.0 (R Core Team, 2025).

## Results

In this six-year follow-up study, asthma remission was strongly associated with disease severity, with lower remission rates observed in patients with severe asthma. Persistent type 2 inflammation, particularly sputum eosinophilia, as well as small airway dysfunction and airflow obstruction, were also linked to reduced remission. Additionally, comorbidities such as dyslipidemia, gastroesophageal reflux disease (GERD), and cardiovascular disease were associated with a lower rate of remission.

This analysis included 203 patients with asthma (mean age, 52 ± 14 years; 45% male; BMI, 27.5 ± 5.7 kg/m^2^; FEV1% predicted, 97.2 ± 21.9; 50% never-smokers). The majority of patients (77%, *n* = 157) had severe asthma (GINA IV-V), while the remaining 46 patients had mild-to-moderate asthma (GINA I-III). Overall, the frequencies of long-term remission, short-term remission, and no remission were 27%, 34%, and 39%, respectively (Table 1).

**Table 1:** Clinical characteristics of asthma patients stratified by remission status and duration. Data are presented as mean ± standard deviation or n (%). Groups include patients with long-term remission (remission sustained for ≥3 consecutive years), short-term remission (remission sustained for 1–2 consecutive years), and non-remission. Sample sizes are indicated at baseline (BL), follow-up 1 (FU1), and follow-up 3 (FU3). Variables include demographic, clinical, lung function, inflammatory markers, treatment, and exacerbation data. Statistical comparisons between groups are shown by p-values. Abbreviations: BMI, body mass index; ICS, inhaled corticosteroids (fluticasone equivalent); OCS, oral corticosteroids; FeNO, fractional exhaled nitric oxide; FEV_1_, forced expiratory volume in 1 s; FVC, forced vital capacity; R5-R20, small airway resistance; AX, reactance area; ACT, asthma control test; IgE, immunoglobulin E

Characteristics	Long-term remission (BL:*n* = 55 FU1:*n* = 55 FU3:*n* = 52)	Short-term remission (BL:*n* = 68 FU1:*n* = 67 FU3:*n* = 52)	Non-remission (BL:*n* = 80 FU1:*n* = 78 FU3:*n* = 57)	p-value
Severe asthmatics (%) BL	45.5	79.4	97.5	<0.001
Age (years) BL	48.7 ± 15.0	52.1 ± 14.3	54.9 ± 11.7	0.04
Sex (% male)	50.1	48.5	37.5	0.2
Ever smoker (%)	45.5	51.5	51.2	0.8
BMI (kg·m^−2^) BL	25.5 ± 3.64	27.7 ± 5.54	28.7 ± 6.68	0.006
BMI (kg·m^−2^) FU1	25.4 ± 3.66	27.9 ± 5.47	28.5 ± 5.74	0.001
BMI (kg·m^−2^) FU3	26.1 ± 3.98	27.7 ± 5.03	28.6 ± 6.09	0.03
Average daily steps BL	9021 ± 3857	7675 ± 2908	7098 ± 3145	0.01
Average daily steps FU1	9156 ± 3376	7608 ± 3199	7433 ± 3761	0.01
Average daily steps FU3	8644 ± 3764	7204 ± 2950	6838 ± 3057	0.15
ICS maintenance (%) BL	83.6	86.8	100	0.001
ICS maintenance (%) FU1	76.4	86.8	100	0.001
ICS maintenance (%) FU3	76.3	84.6	100	0.001
Fluticason equivalent (μg) BL	316 ± 248	629 ± 504	796 ± 523	<0.001
Fluticason equivalent (μg) FU1	300 ± 270	624 ± 494	803 ± 436	<0.001
Fluticason equivalent (μg) FU3	316 ± 248	629 ± 504	796 ± 523	<0.001
OCS (%) BL	0	22.1	45	<0.001
OCS (%) FU1	0	9.0	38.5	<0.001
OCS (%) FU3	0	0	33.3	<0.001
Biological therapy (%) BL	5.5	14.7	17.5	0.1
Biological therapy (%) FU1	5.5	19.4	18.2	0.06
Biological therapy (%) FU3	11.5	41.2	51.8	0.1
Blood eosinophil count (n/μl) BL	0.29 ± 0.19	0.42 ± 0.43	0.33 ± 0.29	0.28
Blood eosinophil count (n/μl) FU1	0.26 ± 0.18	0.33 ± 0.40	0.26 ± 0.27	0.32
Blood eosinophil count (n/μl) FU3	0.24 ± 0.16	0.40 ± 0.48	0.22 ± 0.21	0.06
Sputum eosinophils (%) BL	5.72 ± 12.6	9.17 ± 20.9	13.0 ± 18.3	0.01
Sputum eosinophils (%) FU1	4.25 ± 9.04	6.3 ± 12.2	7.5 ± 12.9	0.5
Sputum eosinophils (%) FU3	2.30 ± 4.84	3.45 ± 6.45	10.3 ± 18.3	0.09
IgE (kU/L) BL	201 ± 277	670 ± 2138	375 ± 736	0.6
IgE (kU/L) FU1	236 ± 229	487 ± 851	704 ± 1290	0.96
IgE (kU/L) FU3	272 ± 413	495 ± 909	286 ± 567	0.28
FeNO (ppb) BL	36.1 ± 45.5	35.0 ± 22.9	44.7 ± 38.2	0.1
FeNO (ppb) FU1	29.9 ± 43.6	33.4 ± 25.4	37.3 ± 37.8	0.04
FeNO (ppb) FU3	26.2 ± 29.0	28.4 ± 20.5	32.5 ± 27.7	0.15
FEV1 (% pred.) BL	92.0 ± 16.6	75.0 ± 20.6	73.9 ± 22.9	<0.001
FEV1 (% pred.) FU1	92.2 ± 14.3	77.9 ± 18.9	75.7 ± 23.1	<0.001
FEV1 (% pred.) FU3	93.1 ± 14.8	76.6 ± 15.7	76.3 ± 26.0	<0.001
FEV1/FVC BL	0.69 ± 0.1	0.60 ± 0.12	0.61 ± 0.13	<0.001
FEV1/FVC FU1	0.69 ± 0.09	0.62 ± 0.11	0.61 ± 0.13	<0.001
FEV1/FVC FU3	0.70 ± 0.09	0.62 ± 0.11	0.60 ± 0.14	<0.001
R5-R20 (kPa/l/s) BL	0.09 ± 0.08	0.16 ± 0.13	0.17 ± 0.15	0.001
R5-R20 (kPa/l/s) FU1	0.08 ± 0.08	0.13 ± 0.09	0.16 ± 0.14	<0.001
R5-R20 (kPa/l/s) FU3	0.08 ± 0.1	0.11 ± 0.1	0.19 ± 0.2	0.005
AX (kPa/l/s) BL	0.71 ± 0.9	1.50 ± 1.5	1.62 ± 1.97	<0.001
AX (kPa/l/s) FU1	0.54 ± 0.71	1.14 ± 1.12	1.55 ± 1.54	<0.001
AX (kPa/l/s) FU3	0.70 ± 0.85	1.04 ± 1.09	1.86 ± 2.35	0.005
ACT score BL	22.6 ± 2.92	18.1 ± 4.31	14.1 ± 5.12	<0.001
ACT score FU1	23.1 ± 1.78	19.6 ± 3.95	15.2 ± 4.53	<0.001
ACT score FU3	23.3 ± 2.15	20.6 ± 3.85	16.4 ± 4.54	<0.001
n exacerbations BL	0.7 ± 1.3	2.1 ± 2.8	3.5 ± 3.5	<0.001
n exacerbations FU1	0.1 ± 0.6	1.0 ± 2.3	2.6 ± 3.4	<0.001
n exacerbations FU3	0.0 ± 0.0	0.2 ± 0.6	1.5 ± 2.1	<0.001

**Fig. 1:**
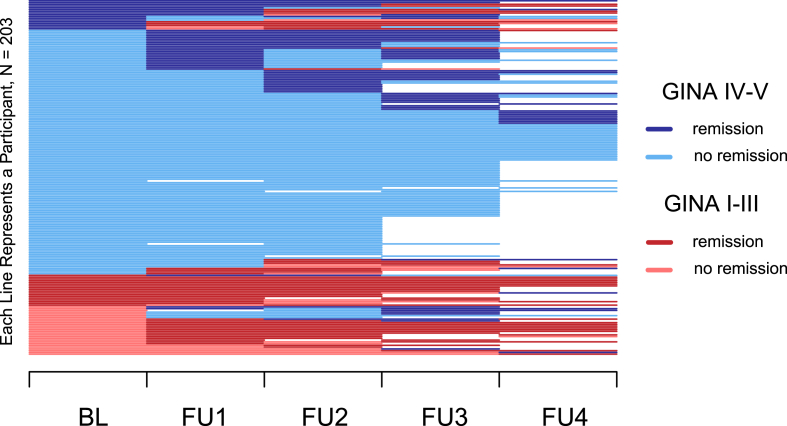
Longitudinal course of asthma remission stratified by GINA treatment steps. The figure depicts individual disease trajectories and treatment intensity over a total follow-up period of 6 years. Each horizontal line represents an individual participant, illustrating changes in asthma remission status and corresponding GINA treatment step across 5 visits: baseline (BL) and follow-ups 1 to 4 (FU1–FU4). Asthma severity was classified according to GINA treatment steps, with mild-to-moderate asthma defined as steps I–III and severe asthma as steps IV–V

The longitudinal course of asthma remission over 6 years, stratified by asthma severity, is illustrated in Fig. 1. Patients with severe asthma had lower remission rates with 50% experiencing at least 1 short-term remission period, and only 16% achieved long-term remission lasting at least 3 consecutive years (Fig. 1). In contrast, patients with mild-to-moderate asthma demonstrated significantly higher remission rates, with 95% experiencing at least 1 short-term remission period and 65% achieving long-term remission (Fig. 1). Among the 73 severe asthma patients who received biological therapy during the six-year follow-up period, 37% achieved remission within the first 12 months of treatment. By 48 months, the remission rate among the same cohort had increased slightly to 39%.

Patient characteristics are shown in Table 1, stratified by remission status at BL, FU1 and FU3. Patients with either no remission or short-term remission were generally older, had higher BMI, and lower levels of physical activity compared to those who achieved long-term remission (Table 1). Despite receiving higher doses of inhaled corticosteroids and more frequent use of systemic corticosteroids, patients with short-term or no remission had significantly elevated T2 biomarkers, particularly sputum eosinophils (Table 1). Furthermore, patients with short-term or no remission had worse lung function, as indicated by lower FEV_1_, increased airflow obstruction on spirometry, and more severe small airway dysfunction, as assessed by oscillometry, with elevated markers of small airway resistance and reactance consistently across all visits (Table 1).

### Predictors of asthma remission

**Fig. 2:**
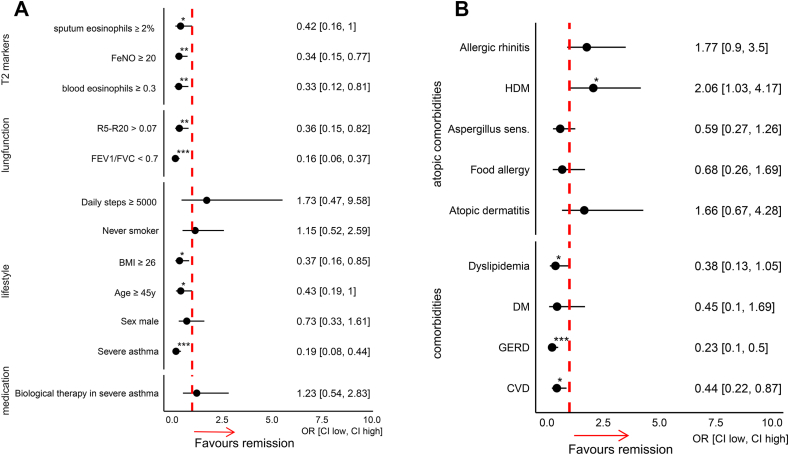
A and B. Forest plots of variables associated with asthma remission. (A) Clinical and biological variables related to asthma remission, including type 2 inflammation markers (T2), fractional exhaled nitric oxide (FeNO), small airway resistance (R5-R20), lung function measures (FEV_1_, FVC), and lifestyle factors. (B) Comorbidities and atopic comorbidities associated with asthma remission, including diabetes mellitus (DM), gastroesophageal reflux disease (GERD), cardiovascular disease (CVD), and sensitization to house dust mite (HDM). Median body mass index (BMI) was 26.3 kg/m^2^ and median age was 52 years. Odds ratios (OR) with 95% confidence intervals (CI) are shown for all variables

In addition to severe asthma, elevated T2-markers and impaired lung function, including both airflow obstruction and small airway disease at baseline visit, were associated with lower remission (Fig. 2A). Furthermore, older age and higher BMI were associated with lower remission rates at the baseline visit. While physical activity, indicated by the number of daily steps, and never-smoking status appeared to be associated with increased remission, neither reached statistical significance. Similarly, biologic therapy in the severe asthma subgroup showed a trend towards increased remission, but this did not reach statistical significance (Fig. 2A).

Among comorbidities, dyslipidemia, gastroesophageal reflux disease, and cardiovascular disease were associated with significantly lower remission rates (Fig. 2B). Regarding allergic conditions, sensitization to HDM was associated with significantly higher remission rates. Allergic rhinitis and atopic dermatitis showed a similar trend towards increased remission, although this did not reach statistical significance (Fig. 2B). Sensitivity to Aspergillus and food allergy were linked to reduced remission, but neither was statistically significant (Fig. 2B).

To account for the longitudinal nature of the data and validate the independent predictors of asthma remission identified through previous analysis, we employed generalized linear mixed-effects models with adjustments for multiple covariates. The model indicated that severe asthma, elevated sputum eosinophilia, small airway dysfunction, and airway obstruction were associated with the absence of asthma remission, independent of age, sex, and BMI (Table 2). Asthma severity and sputum eosinophilia showed the strongest associations, whereas small airway dysfunction and airway obstruction were moderately associated. Data on comorbidities were not available longitudinally and therefore could not be included as predictors in the mixed-effects models. Further analyses explored the role of alternative T2 inflammatory markers, FeNO and blood eosinophils, in asthma remission. The analysis revealed a significant negative association between FeNO levels and asthma remission (supplementary Table E1). However, blood eosinophil levels were not found to be an independent predictor of asthma remission (supplementary Table E2).

**Table 2:** Results of a generalized linear mixed-effects model predicting asthma remission (binary outcome). Fixed effects include standardized variables: asthma severity (GINA treatment step), sputum eosinophil percentage, small airway resistance (R5–R20), airflow obstruction (FEV_1_/FVC), age, sex, and BMI. Random intercepts were included for subject and visit to account for repeated measures. The intraclass correlation coefficients (ICC) were 0.36 for subject and 0.22 for visit. The table reports standard estimates, standard errors, and p-values for each fixed effect. Variance of the random intercepts was 0.80 for subject and 0.46 for visit. Model fit statistics include AIC = 388.2 and BIC = 428.2. The model was fitted with a binomial distribution and logit link

Predictor	Std. Estimate	Std. Error	p-value
GINA step class IV/V	−0.85	0.16	<0.001
R5-R20 (kPa/l/s)	−0.65	0.28	0.02
FEV1/FVC (%)	0.59	0.24	0.02
Sputum eosinophils (%)	−0.70	0.27	0.01
Age (years)	0.03	0.17	0.89
BMI (kg·m^−2^)	−0.36	0.2	0.06
Sex (% male)	−0.23	0.17	0.17
Intercept	−1.27	0.43	0.003

### Remission patterns and clinical determinants by asthma severity

To explore differences in remission profiles between asthma severities, we examined patterns of remission across study visits (Supplementary Fig. 1). In mild-to-moderate asthma (GINA I–III; A), remission was more frequently observed in patients with preserved lung function, lower small airway resistance (R5–R20; B), and fewer T2 inflammatory markers. These features consistently clustered with remission across visits. In contrast, remission was rare in severe asthma (GINA IV–V), where persistent airflow limitation, elevated R5–R20, and multiple positive T2 markers were consistently associated with non-remission.

Regarding clinical remission criteria, the primary reasons for a lack of remission for patients with mild-to-moderate asthma were daily symptoms and impaired lung function (Supplementary Fig. 2A). Patients with severe asthma frequently experienced exacerbations and required the use of oral corticosteroids (Supplementary Fig. 2B).

## Discussion

This cohort study confirms that long-term asthma remission is achievable but appears to be strongly associated with disease severity. Long-term remission occurred in 65% of patients with mild-to-moderate asthma, compared to 16% in those with severe asthma. Among patients receiving biologic therapy, regardless of concomitant medications, 37% achieved remission, consistent with prior reports.^9^^,^^23^^,^^24^ Beyond disease severity, failure to achieve long-term remission was most strongly associated with persistent sputum eosinophilia, while small airway dysfunction and airflow obstruction were moderately associated. Additionally, this study suggests that both atopic and non-atopic comorbidities might be linked to asthma remission.

In our study, failure to achieve asthma remission was associated with persistent type 2 inflammation, particularly elevated sputum eosinophils and FeNO. Notably, participants who did not achieve remission exhibited persistently elevated type 2 inflammatory markers despite high-dose inhaled corticosteroid (ICS) therapy. This observation may point toward ICS non-responsiveness in some patients, where continued high-dose ICS is administered despite only limited clinical improvement. Prior studies indicate that maximal ICS response typically occurs at low-to-medium doses,^22^^,^^23^ while high doses provide little additional benefit and may increase the risk of adverse effects, such as lower respiratory tract infections or major cardiovascular events. These findings highlight the need for individualized assessment of ICS benefit–risk profiles, particularly in patients with persistent T2 inflammation, and support consideration of alternative treatment strategies when remission is not achieved. Moreover, in addition to persistent T2 inflammation, lung function impairment, including small airway dysfunction and airflow obstruction, was associated with the long-term absence of remission. This interplay underscores how sustained T2 inflammation contributes to both persistent lung function impairment and progressive decline.^9^ Consequently, many patients with severe asthma failed to achieve remission primarily due to persistent lung function impairment, even under biologic therapy. This likely reflects the late initiation of treatment, after substantial airway damage has already occurred. These findings highlight the need to reconsider current treatment strategies and support earlier use of biologics to prevent irreversible lung damage.

A critical determinant of lung function in asthma remission is the role of small airway dysfunction. In this study, we demonstrate that oscillometry-defined small airway dysfunction is a strong independent predictor of failure to achieve remission. This finding aligns with previous reports indicating that small airway function is a key determinant of asthma control, providing independent prognostic value beyond airflow obstruction assessed by conventional spirometry.^15^^,^^25^ Moreover, oscillometry offers distinct advantages over spirometry in detecting small airway dysfunction, as its measurements correlate more strongly with both the nature and extent of airway inflammation and more accurately reflect dynamic changes in airway eosinophils.^9^

We investigated the relevance of comorbidities, which may be frequently overlooked contributors to asthma remission. Here, we show that gastroesophageal reflux disease (GERD) is associated with lower rates of asthma remission. GERD is a common comorbidity in asthma, with a significantly higher prevalence in asthma patients than in those without asthma.^26^ While multiple mechanisms link GERD and asthma, the role of T2-inflammation remains less explored. T2-driven esophageal disorders are more frequent in severe asthma (5–10%), and GERD has been associated with persistent symptoms of asthma and a reduced response to anti-eosinophil biologics.^27^ Although asthma often coexists with GERD, their exact relationship is not fully understood, particularly regarding the role of T2-inflammation in esophageal disorders in patients with asthma. Moreover, our findings demonstrate a negative association between cardiovascular disease (CVD), dyslipidemia, and asthma remission. Previous studies have shown that adults with asthma are at an increased risk of developing CVD, with severe exacerbations significantly linked to higher CVD incidence.^28^ A prospective analysis with over 35 years of follow-up identified asthma as a risk factor for CVD, even after adjusting for potential confounders.^29^ Notably, patients who do not achieve asthma remission are often on long-term medium-to-high-dose ICS or OCS. Moderate-to-high daily ICS doses have been associated with an increased but infrequent risk of cardiovascular events, pulmonary embolism, and pneumonia.^24^ Likewise, dyslipidemia has clinical relevance in asthma, as it is associated with specific asthma phenotypes and increased acute exacerbations, independent of other components of the metabolic syndrome.^30^ These findings highlight the importance of evaluating non-atopic comorbidities in asthma management. However, due to the lack of longitudinal data in our cohort, the temporal relationship between these comorbidities and asthma remission could not be assessed. Future longitudinal studies assessing the relationship between comorbidities such as dyslipidemia, GERD, and CVD and asthma remission are warranted.

Lifestyle factors, like physical activity and BMI, play a role in the occurrence of asthma remission. Increased daily steps have been linked to long-term asthma control,^31^ underlining the potential benefits of physical activity. Additionally, a lower BMI has been associated with long-term remission, confirming obesity as an asthma disease modifier.^32^ While not statistically significant, trends suggest that never-smokers may experience better remission outcomes.

### Study limitations

Our study has limitations. First, although the follow-up spanned 6 years, patients were required to attend 3 visits, leading to significant attrition, particularly within the non-remission group, where 26 of the initial 80 patients attended the fourth follow-up. This attrition might have introduced selection bias, as patients experiencing remission may have been less motivated to attend subsequent visits. Second, the study cohort was not evenly distributed, with a higher proportion of severe asthma patients, which may limit generalizability. Third, while biologic therapy varied among subgroup of patients, reflecting clinical practice, this introduced heterogeneity and may affect limited comparability and may affect interpretation of treatment-related outcomes. Fourth, adherence to asthma medication, including inhaled corticosteroids and biologics, was systematically assessed through patient self-report.

## Conclusion

This six-year follow-up study offers a unique perspective on the long-term progression of asthma, revealing the heterogeneous nature of remission and its dependence on factors such as asthma severity, type 2 inflammation, and impaired lung function. The data demonstrate that long-term remission is generally achievable; however, it may remain elusive for many patients, especially in those with severe asthma and persistent T2 markers. These insights highlight a re-evaluation of early interventions targeting type 2 inflammation in order to prevent long-term lung function impairment. Overall, these findings underscore the critical need for personalized management strategies and long-term monitoring to improve understanding of asthma’s trajectory and identify predictors of sustained remission.

## Author contributions

Study conception and design: MA, TB, HW, KFR; conduction of experiment: FP, TB, MA; sample and data collection and analysis: VV, MA, FP, TB; statistical analysis: VV; interpretation of the results: VV and MA. VV and MA drafted the manuscript and all coauthors provided their revision points.

## Authors' consent for publication

The authors declare their consent to the submission and publication of this manuscript and confirm that the final version has been read and approved by all co-authors.

## Availability of data and materials

The datasets used during the current study are available from the corresponding author on reasonable request.

## Ethics approval

The study is a part of the prospective longitudinal All Age Asthma Cohort (ALLIANCE). The study was approved according with the Declaration of Helsinki by the ethics committee at Medical School Luebeck, Germany, and is registered at clinicaltrials.gov (pediatric arm: NCT02496468; adult arm: NCT02419274).

## Statement on the use of AI

During manuscript preparation the use of generative AI-assisted tools was restricted to check spelling and improve readability.

## Funding

The ALLIANCE infrastructure is provided by the participating sites of the German Centre for Lung Research (DZL) and associated study centers and hospitals. Direct costs of the ALLIANCE cohort are being paid by project grants from the German 10.13039/501100002347Federal Ministry of Education and Research (Bundesministerium für Bildung und Forschung, BMBF), grant number “82DZL001A4”.

## Disclosure of conflict of interest

Vera Veith, and Mustafa Abdo report no conflict of interest. Frauke Pedersen, Henrik Watz, Anne-Marie Kirsten, Folke Brinkmann, and Nicole Maison report no relevant conflict of interest. Matthias V. Kopp declares funding from the 10.13039/100009068University of Bern, consulting fees from 10.13039/100004339Sanofi Aventis GmbH and 10.13039/100009946Allergopharma GmbH, honoraria from 10.13039/100004339Sanofi Aventis GmbH, Infectopharm GmbH, and 10.13039/100009946Allergopharma GmbH, participation on an advisory board for 10.13039/100009946Allergopharma GmbH, and a leadership role in the Society of Pediatric Pulmonology. Anna-Maria Dittrich reports grants to institution from Vertex and 10.13039/501100024065OM Pharma, grants from 10.13039/501100002347BMBF and 10.13039/501100001659DFG and personal fees from Glaxo-Smith-Kline, 10.13039/100004336Novartis and Vertex outside of submitted work. Gesine Hansen declares funding from the 10.13039/501100010564German Center for Lung Research (DZL; 10.13039/501100002347BMBF) with grant payments made to 10.13039/501100005624Hannover Medical School, and the 10.13039/501100001659German Research Foundation (10.13039/501100001659DFG), EXC 2155 ‘RESIST’, as well as consulting fees from 10.13039/100004339Sanofi GmbH and lecture fees from MedUpdate and 10.13039/100006483AbbVie. Bianca Schaub reports grants from the 10.13039/501100002347BMBF (German center for lung research, CPC-Munich, 10.13039/501100010564DZL 82DZL033C2, Combat Lung diseases FP4), the German Center for Child and Adolescent 10.13039/100018696Health (DZKJ; 10.13039/501100005722LMU/10.13039/501100005722LMU Klinikum: 01GL2406A), from 10.13039/501100001659DFG (DFG-SCHA 997/8–1 (BS); DFG-SCHA 997/9–1, DFG-SCHA-997/10–1, DFG-SCHA-997/11–1. BS reports consulting fees from 10.13039/100004330GlaxoSmithKline, 10.13039/100004336Novartis, Astra Zeneca, Sanofi; payment/honoraria and participation on a Data Safety Monitoring Board or Advisory Board from 10.13039/100004339Sanofi. Erika von Mutius reports receiving research support, consulting fees, travel funding, honoraria, and patent-related royalties from multiple academic, governmental, and commercial entities, including 10.13039/501100024065OM Pharma S.A., Elsevier, 10.13039/100004325AstraZeneca, and the 10.13039/501100000780European Commission, with no other conflicts of interest declared. Klaus F. Rabe received medical writing support from 10.13039/100019719Chiesi and, in the past 36 months, has received consulting fees, honoraria, or served on advisory boards for 10.13039/100004325AstraZeneca, 10.13039/100008349Boehringer Ingelheim, 10.13039/100019719Chiesi, 10.13039/100004339Sanofi & 10.13039/100009857Regeneron, 10.13039/100008322CSL Behring, 10.13039/100004330GlaxoSmithKline, 10.13039/501100022535Berlin Chemie, and Menarini. Thomas Bahmer received an unrestricted research grant from the 10.13039/501100002347BMBF for the 10.13039/501100010564DZL ALLIANCE cohort, as well as 10.13039/501100002347BMBF funding for NAPKON and 10.13039/501100003107BMG funding for COVIDOM+; in the past 36 months, the author has received consulting fees from Thieme, HealthHero, and Pohl Boskamp, and honoraria from 10.13039/100004319Pfizer, 10.13039/100030732MSD, GSK, 10.13039/100004325AstraZeneca, and 10.13039/100019719Chiesi.
