# Supplementary material for: Exploring trends and predictors of long-term asthma remission
Source: World Allergy Organ J. 2025 Oct 3;18(10):101127. doi: 10.1016/j.waojou.2025.101127 (PMC12528878; doi:10.1016/j.waojou.2025.101127)
Supplement: Multimedia component 1 [file mmc1.pdf]

suppl Figure 1A

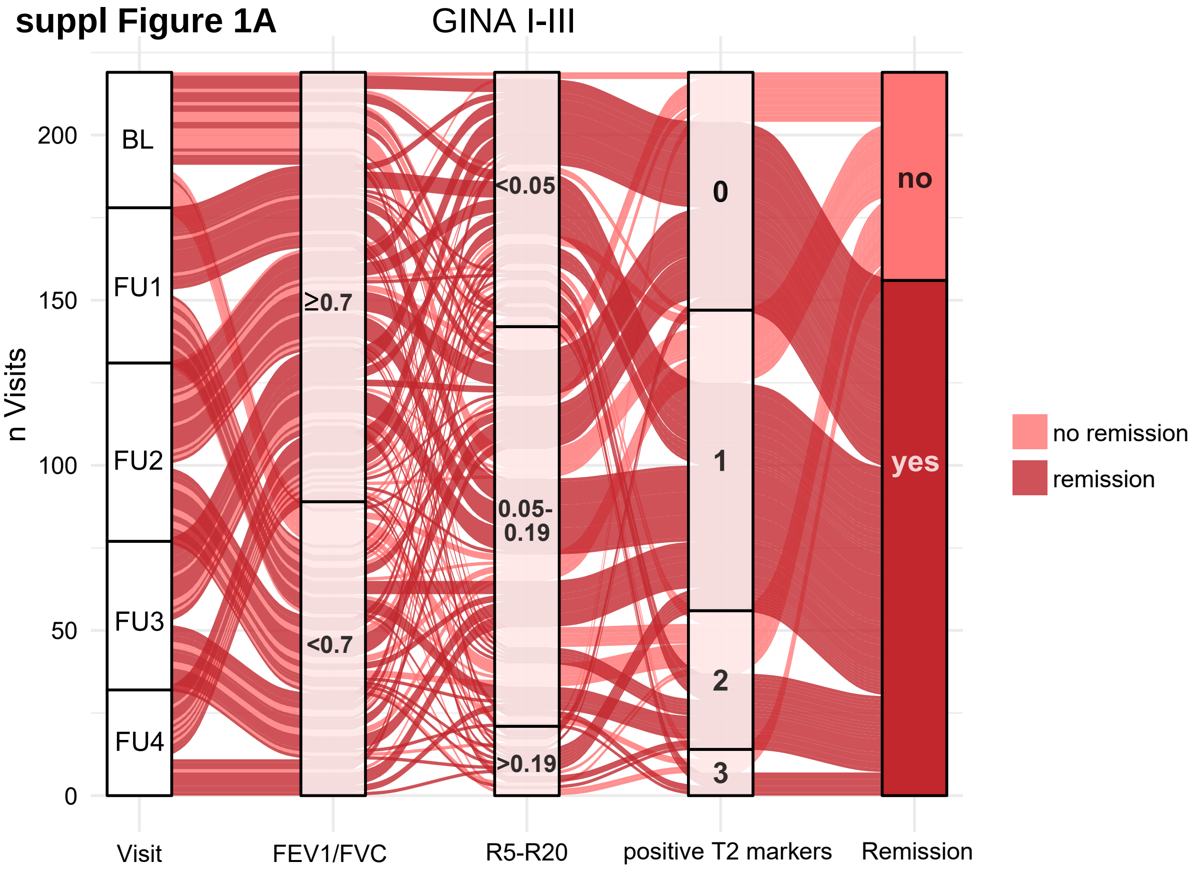

suppl Figure 1B GINA IV-V

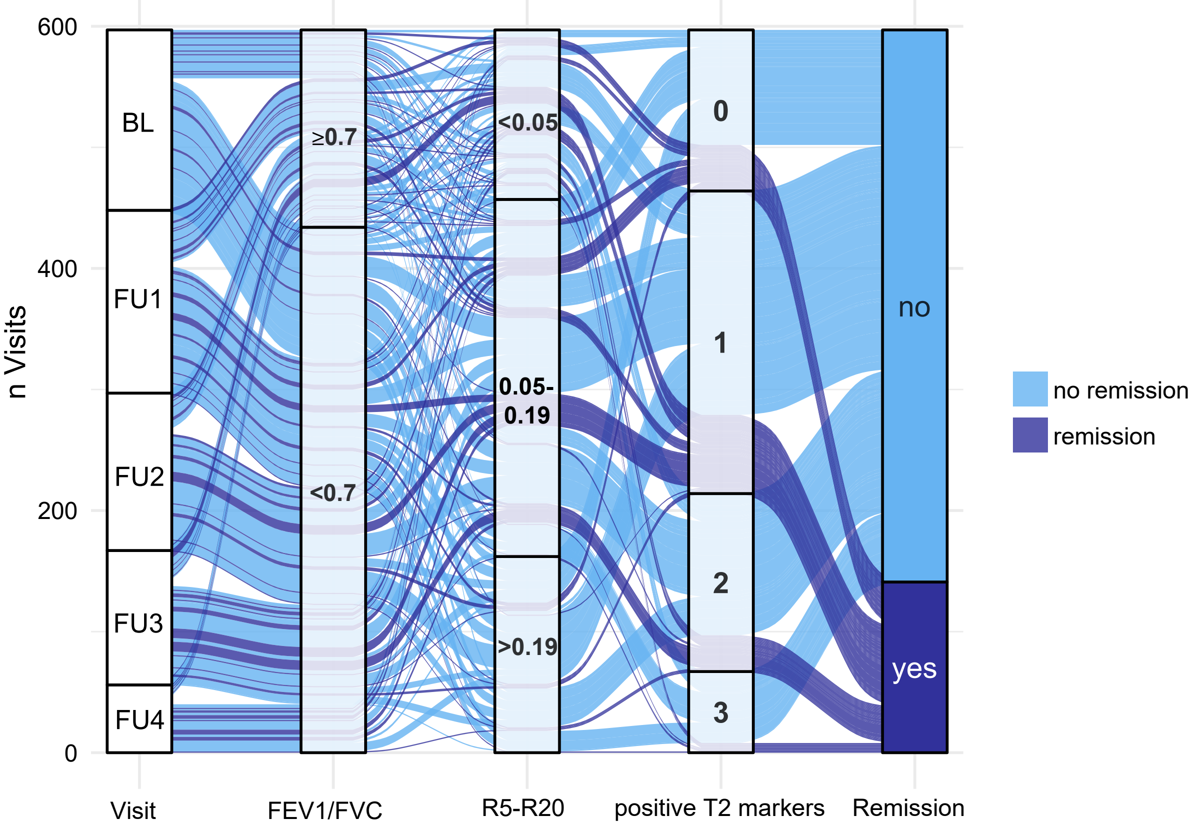

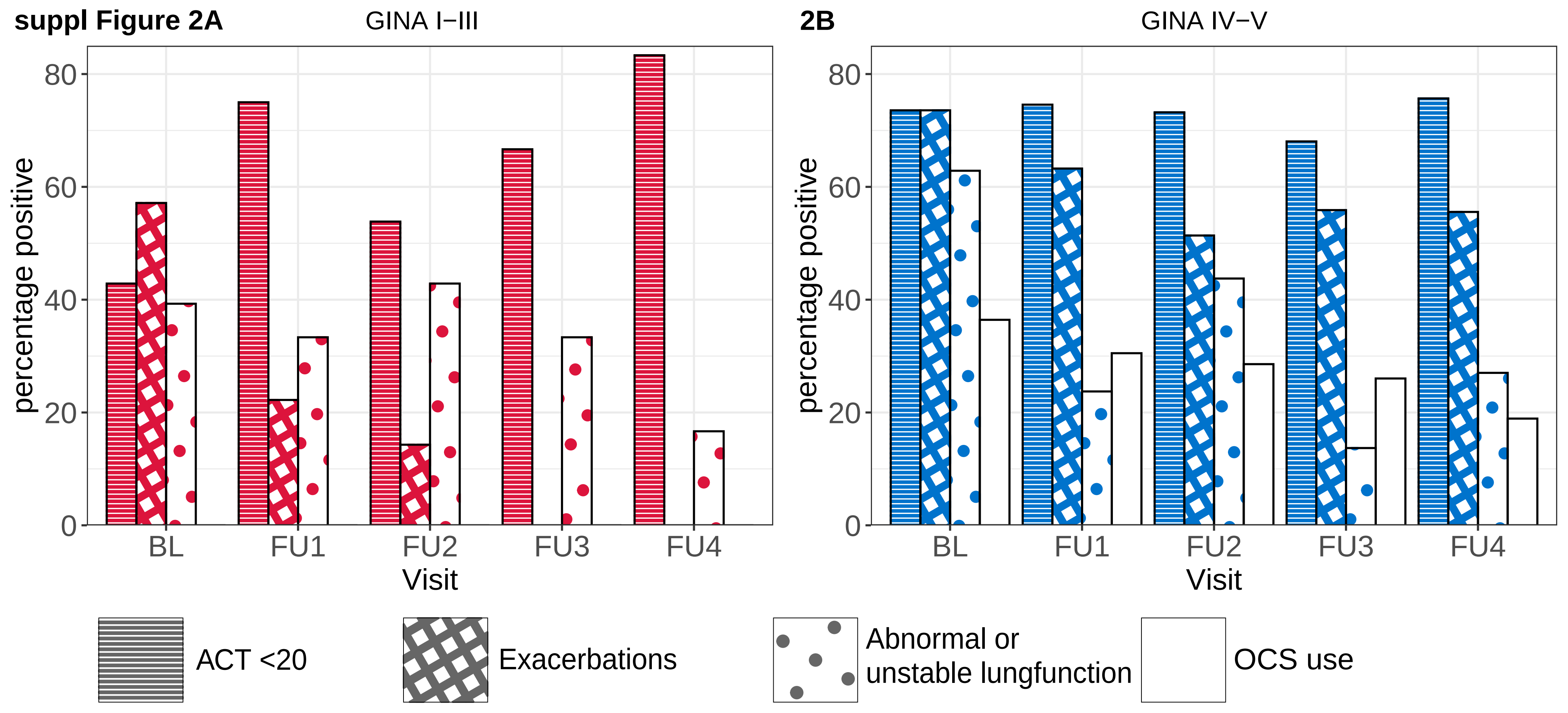
suppl Figure 2A
GINA I−III
2B
GINA IV−V
percentage positive
0
20
40
60
80
BL
FU1
FU2
FU3
FU4
Visit
ACT <20
Exacerbations
Abnormal or unstable lungfunction
OCS use

Supplementary Figure 1: Alluvial plots showing remission patterns in mild-to-moderate (GINA I–III; A) and severe asthma (GINA IV–V; B) based on airflow obstruction (FEV1/FVC), small airway disease (R5–R20), and T2 markers. Remission was more frequent in mild-to-moderate asthma patients with less obstruction, lower R5–R20, and fewer T2 markers, while severe asthma patients with greater obstruction and more T2 markers showed low remission rates.

Supplementary Figure 2:

Failure to achieve asthma remission in mild-to-moderate (GINA steps I–III; A) and severe asthma (GINA steps IV–V; B). Displayed are the four defining criteria for non-remission: daily symptoms, impaired lung function, exacerbations, and oral corticosteroid use. In mild-to-moderate asthma (A), daily symptoms and impaired lung function were the primary contributors, whereas in severe asthma (B), exacerbations and oral corticosteroid use were more common.
