# Supplementary material for: Exploring trends and predictors of long-term asthma remission
Source: World Allergy Organ J. 2025 Oct 3;18(10):101127. doi: 10.1016/j.waojou.2025.101127 (PMC12528878; doi:10.1016/j.waojou.2025.101127)
Supplement: Multimedia component 2 [file mmc2.docx]

Supplementary Tables E1 and E2: Results of generalized linear mixed-effects models predicting asthma remission (binary outcome) stratified by T2 inflammatory markers: fractional exhaled nitric oxide (FeNO, Table E1) and blood eosinophils (Table E2). Fixed effects include scaled variables: asthma severity (GINA treatment step), FeNO or blood eosinophils, small airway resistance (R5–R20), airflow obstruction (FEV₁/FVC), age, sex, and BMI. Random intercepts for subject and visit were included to account for repeated measures.

For Table E1 (FeNO model), random intercept variances were 2.33 for subject and 0.59 for visit (ICC approximately 0.80 and 0.20, respectively). Model fit statistics: AIC = 782.9, BIC = 829.7.

For Table E2 (blood eosinophils model), random intercept variances were 2.31 for subject and 0.64 for visit (ICC approximately 0.78 and 0.22, respectively). Model fit statistics: AIC = 803.4, BIC = 850.5.

E1

| **Predictor** | **Std. Estimate** | **Std. Error** | **p-value** |
| --- | --- | --- | --- |
| GINA step class IV/V | -0.73 | 0.14 | <0.001 |
| FeNO (ppb) | -0.46 | 0.16 | 0.003 |
| R5-R20 (kPa/l/s) | -0.47 | 0.19 | 0.01 |
| FEV1/FVC (%) | 0.58 | 0.20 | 0.003 |
| Age (years) | -0.03 | 0.17 | 0.86 |
| Sex (% male) | -0.35 | 0.16 | 0.03 |
| BMI (kg·m^-2^) | -0.45 | 0.01921 | 0.01 |
| Intercept | -0.9 | 0.39 | 0.01 |
|  |  |  |  |

E2

| **Predictor** | **Std. Estimate** | **Std. Error** | **p-value** |
| --- | --- | --- | --- |
| GINA step class IV/V | -0.72 | 0.14 | <0.001 |
| Blood eosinophil count (/µl) | -61.64 | 146.02 | 0.67 |
| R5-R20 (kPa/l/s) | -0.51 | 0.19 | 0.007 |
| FEV1/FVC (%) | 0.58 | 0.19 | 0.003 |
| Age (years) | -0.02 | 0.17 | 0.9 |
| Sex (% male) | -0.30 | 0.15 | 0.06 |
| BMI (kg·m^-2^) | -0.39 | 0.17 | 0.03 |
| Intercept | -3.11 | 5.06 | 0.54 |
